# Early Diagnosis — Prompt Isolation and Treatment of Colds and Influenza

**Published:** 1918-11

**Authors:** 


### EARLY DIAGNOSIS — PROMPT ISOLATION AND TREATMENT OF COLDS AND INFLUENZA

The early diagnosis of colds and influenza is of very great importance in preventing others from becoming infected; and early treatment, consisting principally of rest in bed, will prevent many cases of secondary streptococcic pneumonia. The early diagnosis also enables the medical officer to isolate promptly the infected soldier, who otherwise is a menace to his comrades.

The common colds and the mild cases of influenza should be regarded as conditions with possibilities of serious complications, and should be treated as promptly and as thoroughly as is done with a mild case of small-pox. The mortality from influenza, when the complications, particularly pneumonia, are included, is perhaps as great as the death rate from small-pox, the epidemics of which have been mild in recent years. A soldier may "catch" pneumonia from the man with a cold, who may be a pneumococcus or streptococcus carrier, just as a man may contract small-pox of the confluent type from a case of varioloid, in which there are only one or two pustules. Of course it is not practicable to isolate every case of influenza when there is an epidemic raging, but the principle of isolation in influenza prevention is sound, and where the best thing cannot be done, the rule should be to approach it as nearly as circumstances will permit.

Company officers should be on the look-out for cases of influenza among their men, and should instruct them to report for treatment with the first symptoms. It is only by co-operation of the line officers with the regimental surgeons that an epidemic of influenza, pneumonia, or any other disease, may be controlled.

In the hospitals, all cases of respiratory infection should be isolated or screened in special wards. Nurses and attendants should wear masks in the wards. Convalescent patients with coughs should still be isolated and should wear masks whenever there is a danger of their coming into contact with others, as in washrooms or at latrines. Examination of the mouths and throats of patients recovering from an attack of pneumonia has shown that they may harbor a virulent type of pneumococcus for from 12 to 90 days after recovery. Attention should be given the mouths and throats of all hospital patients. Since a large percentage of pneumonia cases contaminate the room they occupy, wards should be disinfected with bichloride solution or carbolic acid. In the examination of the dust of a room in which cases of Type 1 and 2 pneumococci had occurred. Stillman found streptococci were present in 74 out of 183 specimens. Pneumococci may remain alive for one to four months in dried sputum. Hence wards should not be swept with a dry broom. Drinking cups and dishes, as well as all towels and linen used by patients, should be carefully disinfected and all sputum burned.

Finally, it seems desirable that a more thorough trial might be given the spray chamber, and that the individuals of those units in which respiratory infection is rife might be passed through such a chamber daily, inhaling the vapor of the antiseptic for about twenty minutes. The favorable results which the New Zealanders obtained in the prevention of respiratory diseases, particularly on their transports, are very encouraging, and it seems very probable that, in the majority of cases at least, the number of pathogenic organisms becomes greatly reduced by such treatment. One litre of the solution is steam-sprayed into a room of approximately 750 cubic feet capacity in the course of 10 to 20 minutes, during which time the individuals remain there inhaling through the nostrils. Chloramine T 2 o/o and zinc sulphate 1.2 o/o have been particularly employed.

